# Efficacy of eltrombopag with immunosuppressive therapy for children with acquired aplastic anemia

**DOI:** 10.3389/fped.2022.1095143

**Published:** 2023-01-10

**Authors:** Yufei Zhao, Wenrui Yang, Xin Zhao, Xiangrong Hu, Jing Hu, Xu Liu, Jianping Li, Lei Ye, Youzhen Xiong, Yang Yang, Baohang Zhang, Xiaoxia Li, Xiawan Yang, Yimeng Shi, Guangxin Peng, Yuan Li, Huihui Fan, Kang Zhou, Liping Jing, Li Zhang, Fengkui Zhang

**Affiliations:** Anemia Therapeutic Centre, State Key Laboratory of Experimental Hematology, National Clinical Research Center for Blood Diseases, Haihe Laboratory of Cell Ecosystem, Institute of Hematology & Blood Diseases Hospital, Chinese Academy of Medical Sciences & Peking Union Medical College, Tianjin, China

**Keywords:** aplastic anemia, bone marrow failure, eltrombopag, hematological response, pediatric, immunosuppressive therapy

## Abstract

**Background:**

Eltrombopag (EPAG), an oral thrombopoietin receptor agonist (TPO-RA), has been proven to improve the hematologic response without increasing toxic effects as a first-line therapy combined with standard immunosuppressive treatment (IST) in adults with severe aplastic anemia (SAA). Nevertheless, the clinical evidence on the efficacy of EPAG in children with acquired aplastic anemia is limited and controversial.

**Methods:**

We performed a single-center, retrospective study to analyze the clinical outcomes of fifteen patients aged ≤18 years with newly diagnosed acquired SAA who received first-line IST and EPAG (EPAG group) compared with those of forty-five patients who received IST alone (IST group) by propensity score matching (PSM).

**Results:**

There was no difference in the overall response (OR) rate between the EPAG group and IST group (53.3% vs. 46.7% at 3 months, *P* = 0.655; 66.7% vs. 57.8% at 6 months, *P* = 0.543), but the complete response (CR) rate was statistically significant (20.0% vs. 4.4% at 3 months, *P* = 0.094; 46.7% vs. 13.3% at 6 months, *P* = 0.012). The median time to achieve a hematological response in the EPAG and IST groups was 105 days and 184 days, respectively. No difference was observed in the event-free survival (EFS) or overall survival (OS) rates.

**Conclusion:**

Adding EPAG to standard IST as the first-line treatment for children with acquired SAA improved the rapidity of hematological response and the CR rate but did not improve the OR or EFS rates.

## Introduction

Acquired aplastic anemia (AA), an immune-mediated bone marrow failure syndrome characterized by hypocellular marrow, pancytopenia, and life-threatening bleeding, infection, and anemia, is mainly caused by cytotoxic T-cell-mediated autoimmune destruction, specifically of bone marrow hematopoietic stem and progenitor cells (HSPCs) (1, 2).

Approaches to the treatment of AA in children and adults primarily include bone marrow transplantation and immunosuppression therapy (3, 4). Immunosuppression therapy (IST), which consists of anti-lymphocyte/thymocyte globulin (ALG/ATG) and cyclosporine (CsA), achieves hematologic responses in approximately 70% of patients (5, 6).

Eltrombopag (EPAG), an oral thrombopoietin receptor agonist (TPO-RA) initially developed for chronic immune thrombocytopenia, was first proven by the National Institutes of Health (NIH) to be effective in severe aplastic anemia (SAA) patients refractory to IST (7), presuming its efficacy in stimulating residual HSPCs. For treatment-naïve SAA patients, EPAG with standard IST was subsequently proven to improve the quality and rapidity of hematologic responses (response rate of nearly 80%) without increasing clonal evolution and toxic effects (8–11).

Studies of EPAG with IST as a first-line therapy have been conducted primarily with adults in the past decade, while clinical evidence for the benefit of adding EPAG to standard IST in children remains limited and conflicting (Supplementary Table S1) (12–20). According to a retrospective study performed by the NIH, it seems that the addition of EPAG to IST did not provide the same benefit in children as that observed in adults with SAA (16). Notably, a multicenter prospective trial recently found that EPAG combined with IST increased the complete response (CR) rate but not the overall response (OR) rate in pediatric patients (12). Due to the controversial results in different studies worldwide, more clinical evidence is needed to ascertain the rational use of EPAG with IST in children with SAA (21). Here, we conducted a single-center, retrospective study to assess the benefit of adding EPAG to standard IST in pediatric patients with SAA by propensity score matching (PSM).

## Methods

We performed a retrospective study on the clinical outcomes of newly diagnosed pediatric patients with SAA (≤18 years old) who underwent IST therapy with EPAG and those who did not receive EPAG as a matched historical cohort in our center. The primary objective was to assess the benefit of adding EPAG to IST as a first-line therapy for children with SAA. The study was approved by the Ethical Committee of the Institute of Hematology and Blood Diseases Hospital, Chinese Academy of Medical Sciences & Peking Union Medical College (IIT2021008-EC-1), and informed consent was obtained from all patients and/or their legal guardians.

### Patients

AA was diagnosed according to the criteria from the International Agranulocytosis and Aplastic Anemia Study (22), while severity was defined by the Camitta criteria and Bacigalupo criteria (23, 24). AA was defined as meeting at least two of the following three criteria for peripheral blood: a hemoglobin (Hb) level <100 g/L, a platelet count <50 × 10^9^/L, an absolute neutrophil count (ANC) < 1.5 × 10^9^/L and a decrease in cellularity in the absence of an abnormal infiltrate or marrow fibrosis on bone marrow biopsy. Patients with inherited bone marrow failure syndromes (IBMFS), paroxysmal nocturnal hemoglobinuria (PNH), and myelodysplastic syndromes (MDS) were excluded through case histories, clinical manifestations, flow cytometry, cytogenetic and fluorescence *in situ* hybridization (FISH) detection, mitomycin C tests, comet assays, and next generation sequencing (NGS) tests.

SAA was defined as bone marrow cellularity <30% and meeting at least two of the following: an ANC <0.5 × 10^9^/L, a platelet count <20 × 10^9^/L, and a reticulocyte count <20 × 10^9^/L. When the ANC was <0.2 × 10^9^/L based on the SAA criteria, it was defined as very SAA (VSAA).

From October 2019 through July 2021, a total of 15 children (aged ≤18 years, EPAG group) were enrolled as patients treated by EPAG and IST in our center. The inclusion criteria were as follows: patients newly diagnosed with acquired SAA; patients who were ineligible for HSCT or voluntarily chose IST; patients treated with EPAG combined with standard IST as a first-line therapy (*p*-ALG/r-ATG and CsA), with no prior IST; and patients with the continuous use of EPAG for more than three months.

To minimize potential confounders and selection bias, we performed a PSM analysis to match patients in the EPAG group with those in the IST group as a historical cohort. From January 2012 through December 2019, 45 children (aged ≤18 years, IST group) were matched from a total of 337 consecutive pediatric patients with newly diagnosed acquired SAA who were treated by standard IST only according to age and severity of disease by PSM. Nearest neighbor matching was performed in a 1:3 ratio with the caliper set at 0.2.

### Treatment

All patients were treated by standard IST, consisting of rabbit-antithymocyte globulin/p-porcine-lymphocyte globulin (r-ATG/p-ALG) and cyclosporine (CsA). Patients received 3 mg/kg/day r-ATG (thymoglobulin, Genzyme) or 20 mg/kg/day p-ALG (Wuhan Institute of Biological Products Co., Ltd) for five consecutive days, with prednisolone (1 mg/kg/day) to prevent serum sickness. CsA was orally administered at an initial dose of 3 mg/kg/day from diagnosis, adjusted to maintain a blood trough level of 150 ∼ 250 *μ*g/L until the best response, and then continued for three months, followed by a slow tapering until discontinuation.

In the EPAG group, patients additionally received eltrombopag (Revolade, Novartis) at a dosage of 2.5 mg/kg/day (the dose was adjusted to match a tablet (25 mg or 50 mg), max dose of 150 mg/day) for at least three months, adjusted according to the platelet counts and tolerance and tapered when the platelet count was >200 × 10^9^/L. EPAG was mostly administered simultaneously with IST.

### Response criteria

Overall response (OR) was categorized as a complete response (CR) or a partial response (PR). A CR was defined as an Hb level ≥100 g/L, a platelet count ≥100 × 10^9^/L, an ANC ≥1.5 × 10^9^/L, and independence of transfusion. A PR was defined as an Hb level ≥70 g/L, a platelet count ≥20 × 10^9^/L, and an ANC ≥0.5 × 109/L, but the blood count criteria for a CR were not met. No response (NR) was defined as blood counts that continued to meet the criteria for SAA. The hematological response was assessed at 3, 6 and 12 months from the first day of ALG/ATG therapy.

Relapse was defined as substantial decreases in blood counts after reaching the best response (PR or CR) and the need for transfusion or second-IST therapy. Overall survival (OS) was measured from the first day of IST until death from any cause or the date of the last follow-up. Event-free survival (EFS) was measured from the first day of IST until any event (lack of response at 6 months, HSCT, death, relapse, repeat IST or any additional AA treatment, transformation to PNH and malignant clonal evolution) or the date of the last follow-up.

### Statistics

All statistical analyses were performed using SPSS 28.0 and R 4.2.1 software. Student's t test was used for continuous variables with a normal distribution and equal variances, the nonparametric Mann‒Whitney U test was used for variables with a significant difference in distribution, and Pearson's chi-squared test or Fisher's exact test was used for categorical variables. Kaplan‒Meier curves were used to compare the cumulative hematological response rate, OS, and EFS, and differences were tested using the Gehan-Breslow-Wilcoxon and log-rank tests. *P* values < 0.05 were considered to indicate significance.

## Results

### Patient characteristics

There were fifteen patients in the EPAG group, including six patients with SAA and nine patients with VSAA, and the median age was thirteen years. There were forty-five patients in the IST group; the basic clinical characteristics of both groups were well balanced (Table 1). No significant differences in age, sex, severity, r-ATG or p-ALG treatment, baseline blood counts, or other main clinical features were observed between the two groups. The median EPAG administration time of the EPAG group was 7.1 (range, 3.3–22.3) months, and the median EPAG administration dose (the first three months) was 62.5 (range, 25–87.5) mg.

**Table 1:** Baseline characteristics of the patients.

Characteristic	Group A Pediatric patients with IST + EPAG (*n* = 15)	Group B Pediatric patients with IST only (*n* = 45)	*P*
Gender (male/female)	10/5	26/19	0.543
Age at treatment (years)			
Median (range)	13 (4∼18)	13 (7-17)	0.700
Severity of aplastic anemia, *n* (%)			1.000
SAA	6 (40)	20 (44.4)	
VSAA	9 (60.0)	25 (55.6)	
History(months)			
Median (range)	1.0 (0.1-8.0)	1.0 (0.2-120.0)	0.567
Baseline laboratory values, median (interquartile range)			
Reticulocyte count (×10^9^/L)	9.2 (3.60-31.60)	7.20 (4.15-18.10)	0.484
Neutrophil count (×10^9^/L)	0.24 (0.06-0.57)	0.18 (0.04∼0.40)	0.393
Lymphocyte count (×10^9^/L)	1.64 (0.73∼2.62)	1.43 (0.44-2.03)	0.398
Platelet count (×10^9^/L)	5 (3∼13)	9 (6-14)	0.364
Hemoglobin (g/L)	66 (55∼83)	61 (55-67)	0.206
Ferritin (ng/ml)			
Median (range)	279.8 (149.8∼2009.0)	360.7 (1.6∼2398.0)	0.645
PNH clones, *n* (%)	3 (20.0)	4 (8.9)	0.351
Treatment of IST, *n* (%)			0.516
rATG + CsA	5 (33.3)	11 (24.4)	
pALG + CsA	10 (66.7)	34 (75.6)	
HSCT after IST, *n* (%)			1.000
Yes	2 (13.3)	5 (11.1)	
No	13 (86.7)	40 (88.9)	
Time between IST onset and EPAG initiation (days)			
Median (range)	0 (-4∼60)	N/A	N/A
EPAG duration (months)			
Median (range)	7.10 (3.33∼22.30)	N/A	N/A
Follow∼up (months)			
Median (range)	19 (7∼34)	74 (1-119)	<0.001

Note: IST, immunosuppressive therapy; EPAG, eltrombopag; SAA, severe aplastic anemia; VSAA, very severe aplastic anemia; PNH, paroxysmal nocturnal hemoglobinuria; CsA, cyclosporine; r-ATG, rabbit-antithymocyte globulin; *p*-ALG, porcine-lymphocyte globulin; HSCT, hematopoietic stem cell transplantation N/A, not available.

### Hematological responses

The hematological responses to IST with or without EPAG in pediatric patients is shown in Table 2. In the EPAG group, the OR rate was 53.3% at 3 months and 66.7% at 6 months compared to that of 46.7% at 3 months and 57.8% at 6 months in the IST group (OR at 6 months, *P* = 0.543), with no significant difference. In the EPAG group, the CR rate was 20.0% at 3 months and 46.7% at 6 months compared to that of 4.4% at 3 months and 13.3% at 6 months in the IST group (CR at 6 months, *P* = 0.012), and patients in the EPAG group achieved a higher CR rate.

**Table 2:** Response to IST with or without eltrombopag in children.

	Group A Pediatric patients with IST + EPAG (*n* = 15)	Group B Pediatric patients with IST only (*n* = 45)	*P*
3 months Response *n* (%)			
Overall Response	8 (53.3)	21 (46.7)	0.655
CR	3 (20.0)	2 (4.4)	0.094
PR	5 (33.3)	19 (42.2)	
NR	7 (46.7)	22 (48.9)	
Off study	0 (0)	2 (4.4)	
6 months Response *n* (%)			
Overall Response	10 (66.7)	26 (57.8)	0.543
CR	7 (46.7)	6 (13.3)	0.012
PR	3 (20.0)	20 (44.4)	
NR	5 (33.3)	14 (31.1)	
Off study	0 (0)	5 (11.1)	
12 months Response *n* (%)			
Overall Response	10 (66.7)	32 (71.1)	0.754
CR	8 (53.3)	12 (26.7)	0.058
PR	2 (13.3)	20 (44.4)	
NR	3 (20.0)	6 (13.3)	
Off study	2 (13.3)	7 (15.6)	

Note: EPAG, eltrombopag; IST, immunosuppressive therapy; CR, complete response; PR, partial response; NR, no response; OR, overall response.

**Figure 1:**
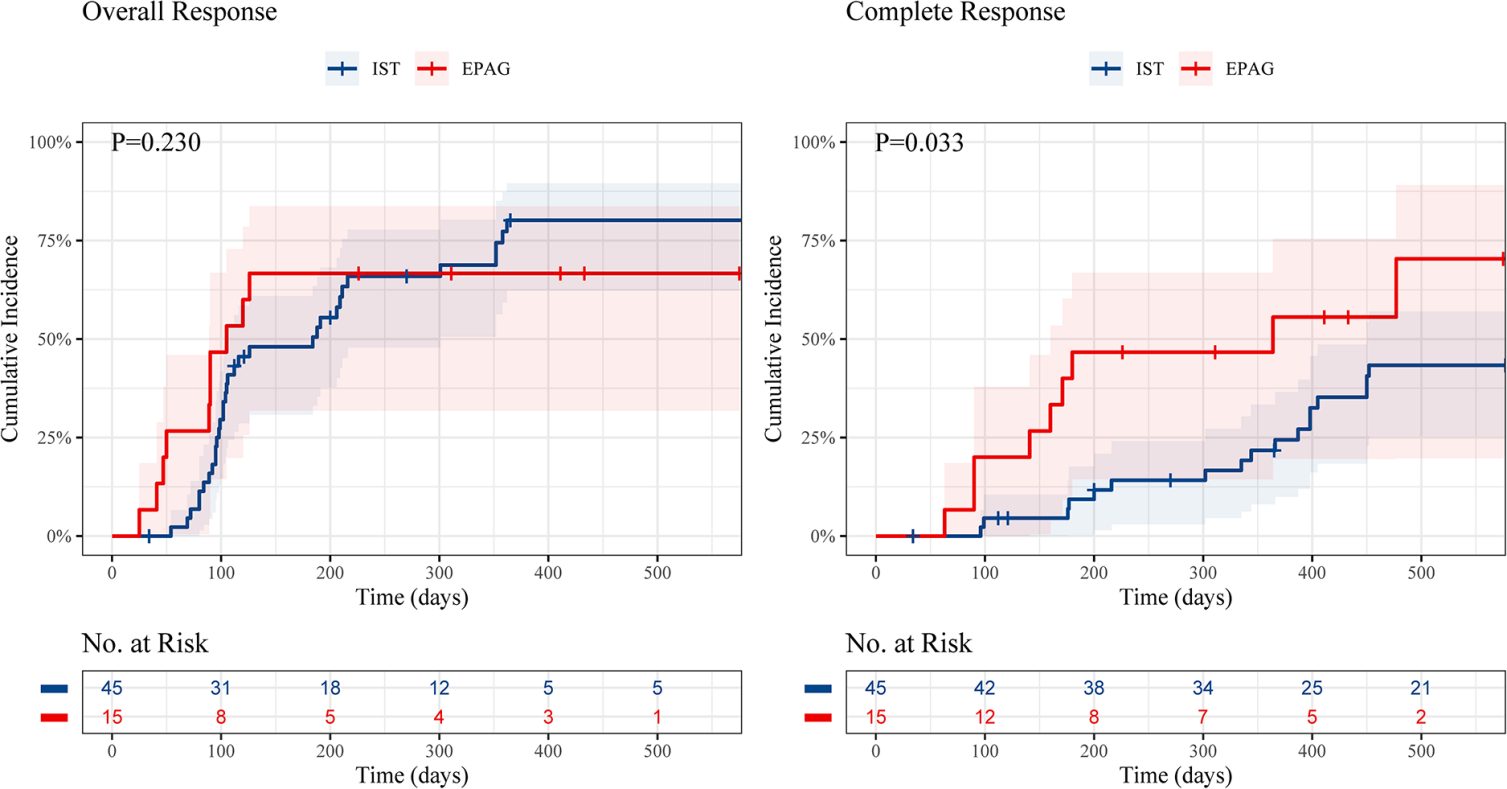
Cumulative incidence of overall response and complete response of children in the EPAG and IST group. EPAG, eltrombopag; IST, immunosuppressive therapy.

The cumulative OR incidence was 66.7% in the EPAG group and 84.1% in the IST group at 18 months (*P* = 0.230), with no significant difference (Figure 1). Nevertheless, the cumulative CR incidence in the EPAG group was statistically higher than that in the IST group, with rates of 70.4% vs. 43.3%, respectively, at 18 months (*P* = 0.033).

Patients in the EPAG group achieved hematologic responses in a shorter period. The median time to an OR in the EPAG group and the IST group was 105 (range, 25–126) days and 184 (range 54–362) days (*P* = 0.008), respectively. In addition, the time to acquire an OR rate of 25% was 50 days vs. 96 days, while the time to acquire a CR rate of 25% was 141 days vs. 387 days in the EPAG group and the IST group, respectively.

From the sixth month to the twelfth month, there was no additional OR (66.7%→66.7%) and only one additional CR (46.7%→53.3%) in the EPAG group, while six patients achieved both an OR (57.8%→71.1%) and a CR (13.3%→26.7%) in the IST group, suggesting faster hematological responses in the EPAG group with less delayed hematological responses.

### Subgroup analysis

According to the median age of thirteen years in our study (Table 3), the OR and CR rates at 6 months in younger children (< 14 years) were 50% (4/8) vs. 70.8% (17/24) (*P* = 0.397) and 12.5% (1/8) vs. 16.7% (4/24) (*P* = 1.000) in the EPAG group and IST group, respectively. For adolescents (≥14 years), however, the OR rate at 6 months was 85.7% (6/7) vs. 42.9% (9/21) (*P* = 0.084), and the CR rate was 85.7% (6/7) vs. 9.5% (2/21) (*P* < 0.001) in the EPAG group and IST group, respectively. The CR rate of the EPAG group was significantly higher in adolescents (≥14 years old).

**Table 3:** Response at 6 months in younger children vs. adolescents.

	Group A Pediatric patients with IST + EPAG	Group B Pediatric patients with IST only	*P*
Younger children (<14 years)	*n* = 8	*n* = 24	
Response *n* (%)			
OR	4 (50)	17 (70.8)	0.397
CR	1 (12.5)	4 (16.7)	1.000
Adolescents (14 years or older)	*n* = 7	*n* = 21	
Response *n* (%)			
OR	6 (85.7)	9 (42.9)	0.084
CR	6 (85.7)	2 (9.5)	<0.001

Note: EPAG, eltrombopag; IST, immunosuppressive therapy; CR, complete response; OR, overall response.

According to the severity of disease (Table 4), for patients with VSAA, the OR and CR rates at 6 months were 55.6% (5/9) vs. 51.9% (14/27) (*P* = 1.000) and 33.3% (3/9) vs. 14.8% (4/27) (*P* = 0.333) in the EPAG group and IST group, respectively, with no significant difference. In contrast, the OR and CR rates at 6 months for patients with SAA were 83.3% (5/6) vs. 66.7% (12/18) (*P* = 0.629) and 66.7% (4/6) vs. 11.1% (2/18) (*P* = 0.018) in the EPAG group and IST group, respectively. In children, patients with SAA had an obviously increased CR rate compared with patients with VSAA.

**Table 4:** Response at 6 months in SAA vs. VSAA.

	Group A Pediatric patients with IST + EPAG	Group B Pediatric patients with IST only	*P*
SAA	*n* = 6	*n* = 18	
Response *n* (%) OR CR	5 (83.3) 4 (66.7)	12 (66.7) 2 (11.1)	0.629 0.018
VSAA	*n* = 9	*n* = 27	
Response *n* (%) OR CR	5 (55.6) 3 (33.3)	14 (51.9) 4 (14.8)	1.000 0.333

Note: EPAG, eltrombopag; IST, immunosuppressive therapy; CR, complete response; OR, overall response; SAA, severe aplastic anemia; VSAA, very severe aplastic anemia.

### Outcomes at the last follow-up

The median follow-up for the EPAG group was 19 (range, 7–34) months and that for the IST group was 74 (range, 1–119) months. Nine patients in the EPAG group (60%) achieved a CR during the follow-up period, and ten patients (66.7%) achieved an OR (Table 5). Two of the patients with NR chose HSCT, and the other three chose additional treatment, such as cyclophosphamide and modifications to avatrombopag or hetrombopag. No patients relapsed during the follow-up time. In the IST group (Supplementary Table S2), three patients died of infections within 6 months, and a total of six patients had relapsed at the last follow-up.

**Table 5:** Treatment outcome of pediatric patients with eltrombopag.

Patients	Gender (F/M)	Age at treatment (years)	Severity (V/SAA)	EPAG duration (months)	EPAG start day (post IST)	EPAG dose (initial, max) (mg)	Follow-up (months)	Response 3M	Response 6M	Response 12M	Latest outcome
1	M	11.1	VSAA	17.2	−4	50,75	29	NR	PR	CR	CR
2	F	9.8	VSAA	5.0	0	50,50	26	NR	NR	Off Study	HSCT 6 months post-IST, died due to complications after HSCT
3	F	16.9	VSAA	6.0	52	75,100	24	NR	NR	NR	Off study
4	M	4.2	VSAA	22.3	42	25,50	24	PR	PR	PR	CR
5	M	16.7	SAA	11.5	−1	50,100	23	PR	CR	CR	CR
6	M	11.0	VSAA	7.0	0	50,75	22	CR	CR	CR	CR
7	M	13.3	SAA	3.3	−1	50,75	22	NR	NR	Off Study	HSCT 8 months post-IST, off study
8	F	10.4	SAA	9.7	−1	12.5,37.5	22	PR	PR	PR	PR
9	M	16.2	VSAA	7.9	0	75,75	20	CR	CR	CR	CR
10	F	11.6	VSAA	6.2	−2	25,25	19	NR	NR	NR	Off study
11	M	15.5	SAA	7.1	1	75,75	19	PR	CR	CR	CR
12	M	12.9	VSAA	5.5	−3	50,50	13	NR	NR	NR	Off study
13	M	17.6	SAA	8.3	0	75,75	16	PR	CR	CR	CR
14	F	18.0	VSAA	12.0	60	50,75	27	NR	CR	CR	CR
15	M	14.3	SAA	4.0	20	25,75	34	CR	CR	CR	CR

Note: EPAG, eltrombopag; SAA, severe aplastic anemia; VSAA, very severe aplastic anemia; IST, immunosuppressive therapy; HSCT, hematopoietic stem cell transplantation; CR, complete response; OR, overall response; PR, partial response; NR, no response.

**Figure 2:**
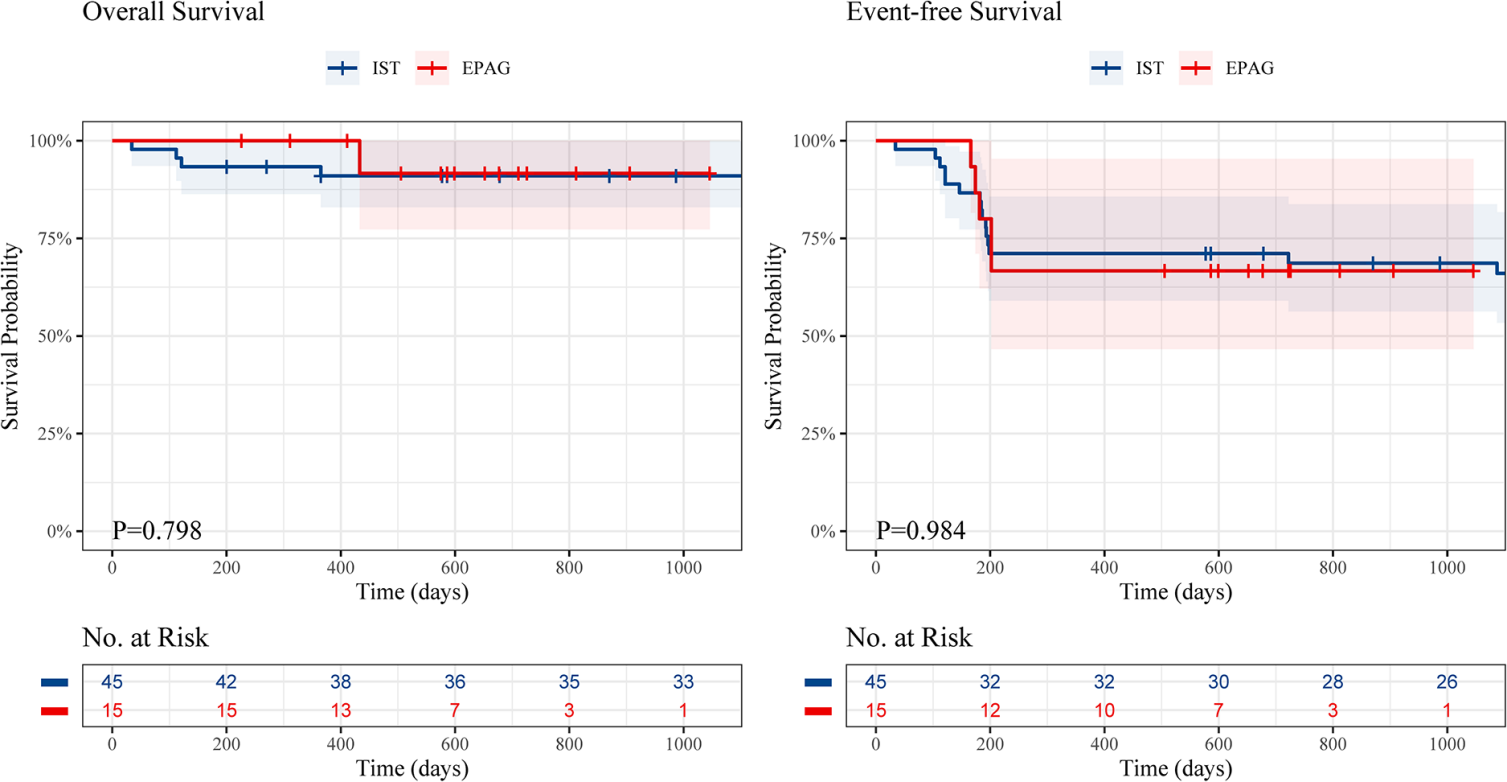
The overall survival and event-free survival of children in the EPAG and IST group. EPAG, eltrombopag; IST, immunosuppressive therapy.

The event-free survival (EFS) rate was similar in the EPAG group and IST group (1.5-year EFS, 66.7% vs. 71.1%, *P* = 0.984) (Figure 2), and no difference was found in the overall survival (OS) rate of the groups (1.5-year OS, 91.7% vs. 91.0%, *P* = 0.798).

Eltrombopag was well tolerated, with no serious adverse events observed related to the therapy. Five patients in the EPAG group had grade 2 hyperbilirubinemia, and one patient showed grade 2 liver test abnormalities.

## Discussion

The clinical evidence that adding EPAG to standard immunosuppressive therapy improves the rate, rapidity and strength of hematologic responses in treatment-naïve patients with SAA is mainly based on adults. The clinical studies on children are limited, most of which were retrospective noncontrolled studies with small sample sizes, with only one prospective study (Supplementary Table S1), and the results are conflicting. We retrospectively analyzed the clinical outcomes of fifteen children with acquired SAA who received IST + EPAG and forty-five patients who were treated by IST alone by PSM, which may better reflect the real results.

Groarke et al. presented a retrospective subgroup analysis of EPAG plus standard IST as a first-line therapy for pediatric patients with SAA (aged <18 years) enrolled in the NIH study (16), which showed no significant improvement in either the OR or CR rates at 6 months or EFS compared with a historical IST cohort. Other retrospective studies, however, showed a better hematologic response with EPAG in children, consistent with the results in adults. Recently, Goronkova et al. reported a randomized prospective multicenter trial to compare the efficacy of EPAG combined with IST and IST alone in treatment-naïve children with SAA (12), which showed that the OR rate at 4 months in both groups was similar (65% vs. 53%), but the CR rate in the EPAG group was significantly higher (31% vs. 12%). In our study, compared with that of the historical cohort matched by PSM, the OR rate at 6 months in the EPAG group was similar (66.7% vs. 57.8%), and a higher CR rate was found in the EPAG group (46.7% vs. 13.3%), which indicated that adding EPAG could improve the quality of hematological responses in children.

In our study, EPAG did not statistically increase the OR or EFS rate in children with SAA. We previously observed an OR rate of approximately 70% in the group of children who received standard IST (25, 26), among which some patients who did not achieve a CR may have had an appropriate amount of residual HSPCs that could be stimulated by EPAG and thus advance to CR. The other 30% of patients who did not achieve an OR may have only a small number of HSPCs or other unclear mechanisms of pathogenesis, making it difficult for improvement by TPO-RAs, and only a small number of patients could benefit from EPAG. Therefore, a larger sample size is required to determine whether added EPAG leads to an increased OR rate in 30% of children with NR.

Patients with SAA present with severe bone marrow failure, pancytopenia, and a high risk of fatal adverse events such as bleeding and infection. Therefore, faster hematological responses and earlier recovery from dangerous situations should be important goals as well. In our study, the median time to reach an OR was 105 days vs. 184 days in the EPAG group and IST group, suggesting that the addition of EPAG resulted in an earlier prevention of the risk of mortality. In addition, the hematologic response in the EPAG group leveled off after the sixth month, while a small portion of patients in the IST group continued to achieve a response from the sixth to the twelfth month, indicating that the addition of EPAG could lead to a more rapid hematologic response in children.

It is acknowledged that patients who did not achieve an OR at four or six months after IST should receive HSCT or secondary IST. In our IST group, patients still had an obvious delayed hematological response after the sixth month, making it difficult to identify patients who may have a delayed response or those who are still at risk. In our EPAG group, however, few patients had delayed responses, suggesting that for patients who continued to have NR at the sixth month after starting EPAG and IST treatment, immediate salvage treatment could be considered to eliminate the risk. Therefore, EPAG may help reduce the number of patients with delayed hematological responses to unnecessary secondary treatments, which helps us make a more precise therapeutic strategy.

Groarke et al. reported that younger children (<12 years) had lower response rates than adolescents with the addition of EPAG (16), with OR and CR rates of 63% vs. 78% and 6% vs. 46%, respectively. In our study (Table 3), consistent with the previous study, younger children (<14 years) had lower response rates than adolescents, with an OR rate of 50.0% (4/8) vs. 85.7% (6/7) and a CR rate of 12.5% (1/8) vs. 85.7% (6/7), respectively, indicating that younger children may not benefit from EPAG. Limited by the small sample size, the conclusion needs to be further verified, and it is necessary to explore the potential mechanism underlying the difference in responses in younger children and adolescents.

The prospective study reported by Goronkova et al. showed that EPAG combined with IST increased the OR rate in pediatric patients with SAA but not in those with VSAA (12). In SAA patients, the OR rate at 4 months was significantly higher in the EPAG + IST group than in the IST group (89% vs. 57%) but this was not observed in VSAA patients (52% vs. 50%). In our study (Table 4), for patients with SAA, the OR and CR rates at 6 months were 83.3% (5/6) vs. 66.7% (12/18) in the EPAG group and the matched IST group, respectively, showing an increasing trend with EPAG, and the CR rate of the EPAG group was significantly higher than that of the IST group (66.7% (4/6) vs. 11.1% (2/18)). For patients with VSAA, however, there were no significant differences in the OR and CR rates between the EPAG group and the IST group at 6 months, consistent with the results of the prospective study. Consequently, patients with relatively more residual HSPCs may have an obviously increased quality of response from the addition of EPAG.

For the past few years, with the development of supportive care, patients with AA could achieve better long-term survival. We found that there were no deaths within the first year in the EPAG group, while there were three patients in the IST group who died of infection within 6 months, which, to some extent, suggests that the addition of EPAG could help prevent early severe conditions by stimulating residual HSPCs.

EPAG was well tolerated, with no serious adverse events. Limited by the short follow-up period, there were no patients who relapsed in the EPAG group at the last follow-up.

The limitations of this study include that it was a retrospective study in a single institution, with a small sample size and short follow-up period, as well as the difference in the duration of EPAG treatment and the interval between EPAG and IST. The median age of patients in the EPAG group was relatively older; thus, we lack clinical data for young children. Nevertheless, we used PSM analysis to match a historical comparison cohort of consecutive patients with SAA to minimize potential confounders and selection bias, and this real-life clinical experience showed comparable results to those reported in the prospective study mentioned above. Furthermore, it is noteworthy that the result of a phase 2 study of EPAG in pediatric patients with previously untreated SAA from Novartis (NCT03025698) is forthcoming, which may serve as strong evidence on the definitive value of additional EPAG to IST.

In conclusion, adding EPAG to standard IST as a first-line treatment for children with acquired SAA increased the quality and rapidity of hematological responses but did not improve the overall response. By promoting early responses, EPAG shortens the duration of life-threatening severe cytopenia and thus could help reduce the risk of potential bleeding, infection, and anemia. A larger cohort with longer follow-up is required to further assess response durability. In addition, the ongoing prospective clinical trials and real-life experiences in pediatric centers are expected to ascertain the role of EPAG in children and further confirm the results of our current study.

## Data Availability

The original contributions presented in the study are included in the article/**Supplementary Material**, further inquiries can be directed to the corresponding author/s.
